# Defining failure is not enough

**DOI:** 10.1038/s41433-026-04601-2

**Published:** 2026-06-06

**Authors:** Mark H. Nelson

**Affiliations:** Retinanet, 4205 Milhaven Lake Court, Winston-Salem, NC USA

**Keywords:** Predictive markers, Biomarkers

The concept of anti-VEGF treatment failure in Neovascular Age-Related Macular Degeneration (nAMD) is widely used in both clinical practice and the literature. Terms such as tachyphylaxis, treatment resistance, persistent fluid, and poor response are routinely applied to eyes that fail to fully suppress exudation, recur early, or remain difficult to extend. Standardising this terminology is necessary [1], however, it does not explain failure itself. The central question is whether recurrent or persistent exudation reflects incomplete suppression of a relevant pathway or a mismatch between therapy and the biological processes sustaining leakage during treatment.

Persistent or recurrent exudation is a shared clinical endpoint, but not a shared mechanism. Eyes that appear to “fail” in similar ways may differ fundamentally in their underlying choroidal neovascularisation (CNV) biology, spanning states from persistent VEGF dependence to structural, inflammatory, or hemodynamic drivers of leakage. If all recurrence is attributed to pharmacologic wear-off, variability becomes a problem of dosing. If, instead, recurrence reflects residual disease activity during therapy, then patterns of persistence and variability in durability become clinically interpretable signals of underlying biology.

Among treated eyes, some achieve complete suppression with predictable recurrence intervals, whereas others recur early or fail to fully dry despite continued therapy. In practice, these patterns are managed rather than interpreted. Treatment intervals are adjusted, doses escalated, and agents switched without fully considering what these response patterns reveal. CNVs that never fully suppress are not biologically equivalent to those that suppress and then recur predictably, suggesting that variability in durability reflects distinct states of residual disease activity during therapy.

Durability is not simply an outcome, but a functional phenotype reflecting the likelihood of recurrence during therapy, and therefore a clinically accessible proxy for residual disease activity. Interpreting durability in this way requires a framework that links treatment behaviour to mechanism. I refer to this approach as Mechanism-Matched Endotypes (MME), in which neovascular lesions are understood in terms of dominant biological pathways together with structural and hemodynamic modifiers. The framework organises clinically observable patterns into biologically interpretable states rather than fixed disease categories [2].

A central implication of this framework is that visible exudation is a threshold event, not a direct measure of biologic activity. Leakage may be present across a range of underlying biological activity and becomes clinically detectable only when compensatory mechanisms are exceeded and fluid accumulates. Optical coherence tomography (OCT), therefore, represents an indirect, threshold-dependent readout of disease activity, influenced by detection thresholds, visit frequency, and retreatment criteria. Observed durability is not a direct measure of activity, but a sampled manifestation of it.

Within this context, therapy functions as a biological probe. Each treatment is a perturbation, defined as a controlled intervention that modulates specific biological pathways within the lesion. The response to that perturbation reveals which pathways remain active during therapy. Persistent or recurrent exudation is therefore not simply treatment failure, but evidence of residual mechanisms not fully addressed by the therapeutic action being applied.

Patterns of response under perturbation can be informative. Durable suppression with anti-VEGF therapy suggests continued VEGF dependence, whereas incomplete suppression or early recurrence suggests activity outside the VEGF axis or incomplete pathway suppression. Extension of durability with dual-pathway inhibition, as demonstrated in TENAYA and LUCERNE [3], is difficult to explain by pharmacologic exposure alone and supports a role for endothelial destabilisation in sustaining leakage during therapy, consistent with prior work implicating Angiopoietin 2 (Ang-2) – mediated vascular instability [4]. Structural response to photodynamic therapy may indicate lesions driven by aneurysmal or more mature vascular configurations, as demonstrated in EVEREST II [5].

Exudation during therapy is an endpoint, not a mechanism. Similar OCT appearances may conceal fundamentally different biological states with distinct therapeutic vulnerabilities. CNVs that appear similar at a single time point may behave very differently over time, with durability emerging as a clinically observable sign of underlying biology. When therapy aligns with the dominant pathway, suppression may be complete and durable. When alignment is partial or absent, recurrence may occur earlier or persist despite continued treatment.

Reframing treatment response in this way has several implications for clinical practice. Persistent or recurrent exudation should not be interpreted uniformly as treatment failure, but as a sign of the underlying mechanism. Durability may guide therapeutic selection by distinguishing CNV that remain controlled with VEGF-directed therapy from those that reflect pathway mismatch and which may benefit from alternative or adjunctive strategies. Treatment switching becomes not only a management decision but a biologically informative intervention, with changes in response providing inferential, though not definitive, evidence of pathway dependence.

Importantly, misinterpretation of underlying biology may also lead to a different form of failure. The decision to escalate VEGF suppression assumes continued pathway dependence. However, VEGF provides trophic support to the retina and choriocapillaris, and in lesions where it is no longer the dominant driver, continued suppression may contribute to atrophic progression [6].

These concepts also have implications for clinical trials. Treating nAMD as a biologically homogeneous condition risks diluting pathway-specific effects. Incorporating response patterns, durability metrics, and therapeutic perturbation into trial design may better capture biologically relevant differences between therapies. Integration of biomarker measurements with defined perturbations may further enable direct assessment of target engagement and downstream response, supporting a shift toward mechanism-informed retinal therapeutics.

In current practice, variability in treatment response is managed pragmatically but rarely interpreted biologically. The MME framework proposes that this variability is not incidental, but informative. Differences in suppression, durability, and recurrence reflect distinct states of residual disease activity during therapy. Until variability in treatment response is interpreted biologically rather than described operationally, recurrence will continue to be managed, but not understood.
